# Burden of hospitalizations over time with invasive aspergillosis in the United States, 2004–2013

**DOI:** 10.1186/s12889-019-6932-9

**Published:** 2019-05-17

**Authors:** Marya D. Zilberberg, Rachel Harrington, James R. Spalding, Andrew F. Shorr

**Affiliations:** 1 EviMed Research Group, LLC, PO Box 303, Goshen, MA 01032 USA; 2 Astellas Pharma Global Development, Inc., Northbrook, IL USA; 3 Washington Hospital Center, Washington, DC USA

**Keywords:** Population study, HCUPnet, United States, Hospitalizations, Invasive aspergillosis, Mortality, Cost

## Abstract

**Background:**

Using aggregated data available on the interactive website from the Agency for Healthcare Research and Quality’s Healthcare Cost and Utilization Project Network (HCUPnet), we examined the annual volume of invasive aspergillosis (IA)-related hospitalizations in the US.

**Methods:**

This was a population study. Age-adjusted volumes were derived through population incidence calculated using year-specific censal and intercensal US population estimates available from the US Census Bureau. We additionally examined IA as the principal diagnosis and its associated outcomes in patients with ICD-9-CM codes 117.3, 117.9 and 484.6.

**Results:**

The age-adjusted number of annual hospitalizations with IA grew from 35,968 cases in 2004 to 51,870 in 2013, a 44.2% overall increase, 4.4% per annum. Regionally, the South contributed the plurality of the cases (40%), and the Northeast the fewest (17%). While IA as principal diagnosis dropped, from 14.4 to 9.3%, mortality rose from 10 to 12%. Despite mean hospital length of stay decreasing from 13.3 (standard error [SE] 0.07) to 11.5 (SE 0.6) days, the corresponding mean hospital charges rose from $71,164 (SE $5248) to $123,005 (SE $9738). The aggregate US inflation-adjusted hospital charges for IA principal diagnosis rose from $436,074,445 in 2004 to $592,358,369 in 2013.

**Conclusions:**

Given the substantial volume and rate of growth in IA-related hospitalizations in the US between 2004 and 2013, an increase in mortality and high costs, IA may represent an attractive target for intensive preventive efforts.

## Background

Invasive aspergillosis (IA) represents a severe infection in many types of patients. It is a growing problem, given the broader use of intensive immunosuppressive regimens for various diseases, increases in organ transplantation and improved diagnostics [1, 2]. Recent US studies described a substantial increase in the prevalence of IA in general, and specifically among patients with hematologic malignancies and those undergoing organ transplants [3]. Despite aggressive prophylaxis in select populations, IA remains associated with high crude and attributable mortality, and by some estimates, the economic costs of IA approach $600 million annually in the US [4].

Limited data exist, however, describing in detail recent trends in epidemiology and outcomes associated with IA. Hence, we aimed to explore time trends in the prevalence, mortality and hospital resource utilization associated with IA among a diverse cohort of hospitalized patients.

## Methods

We estimated the annual burden of all IA-related hospitalizations in the US between 2004 and 2013 using similar methods to those published previously [5–15]. The data for this analysis were derived from the HCUPnet, a website for querying aggregate data from the Healthcare Cost and Utilization Project (HCUP), administered by the Agency for Healthcare Research and Quality (AHRQ) [16]. Specifically, we examined the annual incidence of IA-related hospitalizations, based on the presence of the International Classification of Diseases, version 9, Clinical Modification (ICD-9-CM) codes 117.3, 117.9 and 484.6, which were shown to be sensitive in detecting IA [17]. We examined population incidence (reported as cases per 100,000 population) using year-specific censal and intercensal US population estimates available from the US Census Bureau [18]. We additionally examined IA incidence by normalizing it to all hospitalizations within a given year (reported as cases per 10,000 all-cause hospitalizations). Each incidence estimate was stratified by age group and census region in separate analyses.

We also estimated the annual burden of hospitalizations with IA as the principal discharge diagnosis. IA as the principal diagnosis is presented as a proportion of total IA hospitalizations per annum. On the assumption that principal diagnosis implies that the outcomes of the hospitalization could be attributed to IA, we examined unadjusted mortality, length of stay (LOS), costs and charges (both raw and inflation-corrected) in this cohort. To correct costs and charges for inflation, we employed the medical component of the Consumer Price Index available from the US Bureau of Labor Statistics [19]. The variation in these outcomes was assessed using standard error (SE) estimates where available. No formal inferential statistical testing was undertaken.

## Results

**Fig. 1:**
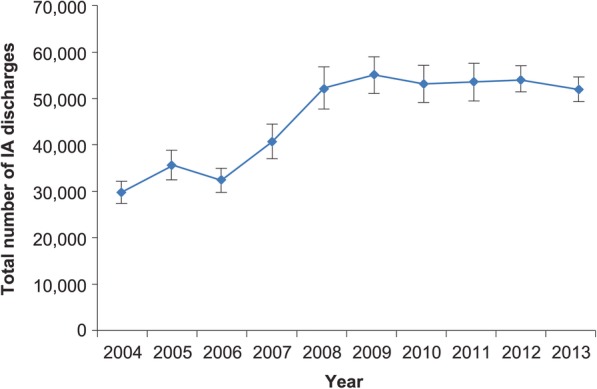
Overall annual volume of hospitalizations with invasive aspergillosis. IA, invasive aspergillosis; vertical bars represent standard error

**Fig. 2:**
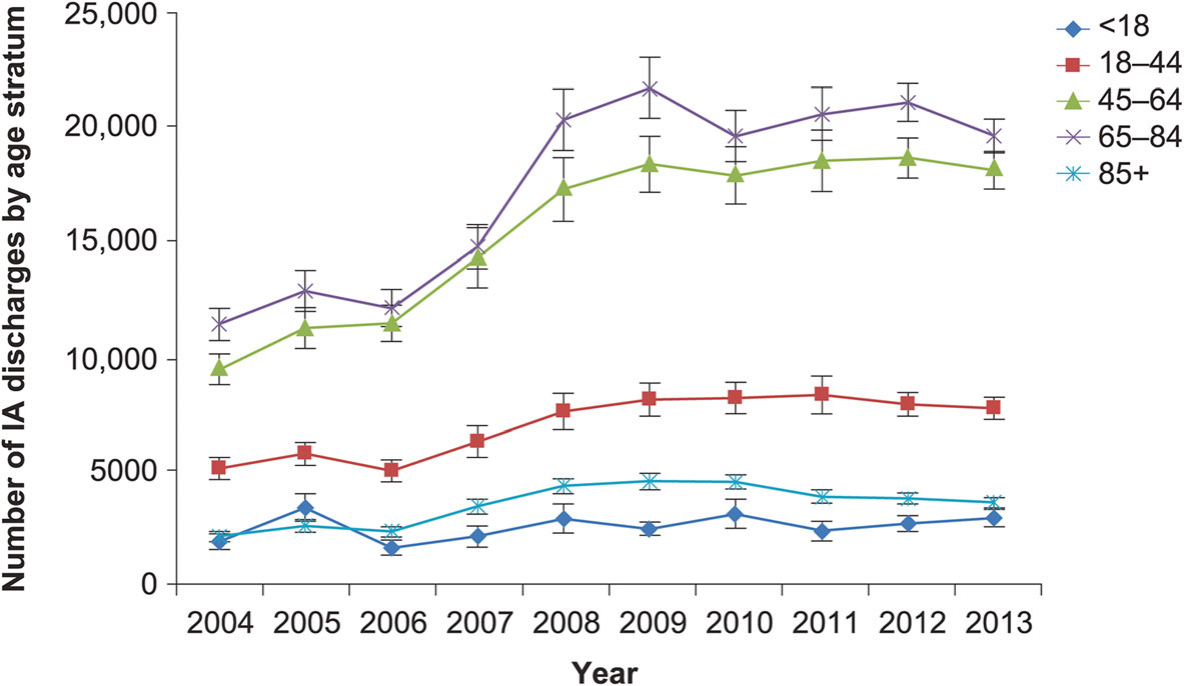
Annual volume of hospitalizations with invasive aspergillosis, by age stratum. IA, invasive aspergillosis; vertical bars represent standard error

**Fig. 3:**
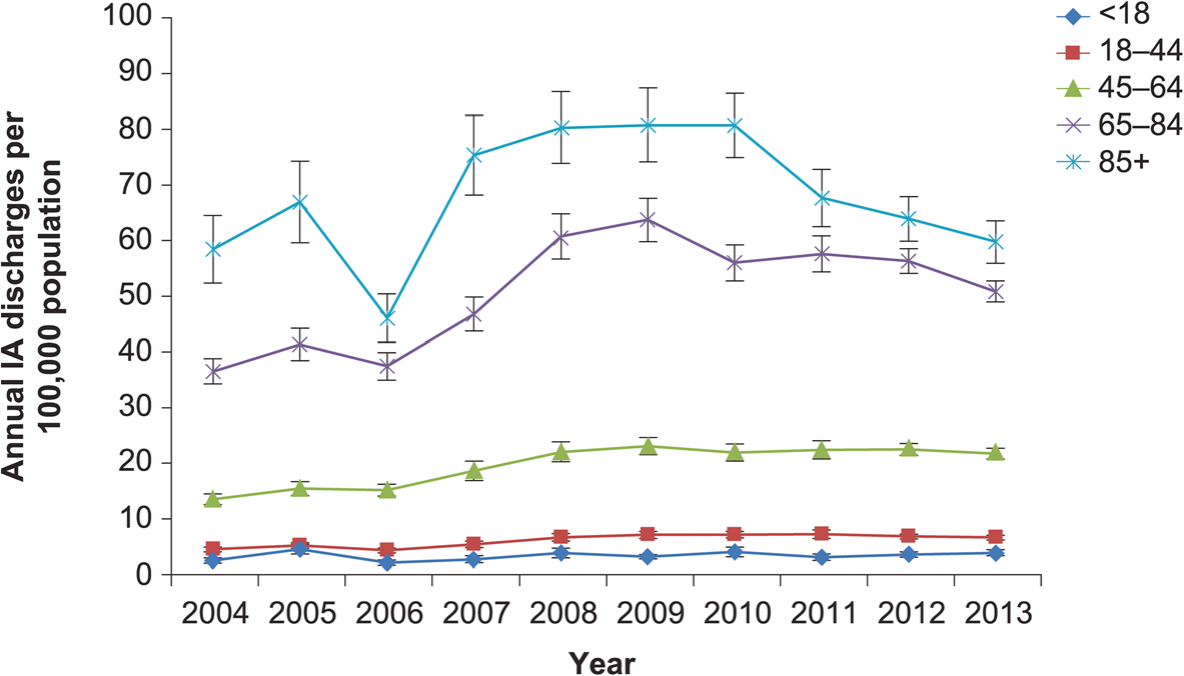
Annual incidence of hospitalizations (per 100,000 population) with invasive aspergillosis, by age stratum. IA, invasive aspergillosis; vertical bars represent standard error

Between years 2004 and 2013, the total volume of annual hospitalizations related to IA rose from 29,774 (SE 2425) to 51,870 (SE 2642), a 74.2% overall growth (Fig. 1). When stratified by age, the highest volume, persisting over time, was observed in those aged 65–84 years, followed by the 45–64-year-old cohort (Fig. 2). The steepest growth occurred between 2006 and 2008 (Fig. 2). Fluctuations in discharges were more pronounced in the age-stratified incidence (Fig. 3). Notably, across all age strata, the annual incidence of hospitalizations with IA was directly proportional to age. Thus, the age group 85+ years exhibited the highest annual incidence of IA hospitalizations throughout the study timeframe. Their IA incidence fell from 58.47 cases per 100,000 population in 2004 to the low of 46.12 in 2006, rising again to the high of 80.82 cases in 2008, and subsequent falling again to 59.78 in 2013 (Fig. 3). When age-adjusting the overall population incidence of IA hospitalizations to 2013, the growth in the volume of cases is more modest, yet still substantial, going from 35,968 cases in 2004 to 51,870 in 2013, a 44.2% growth, or 4.4% per annum.

**Fig. 4:**
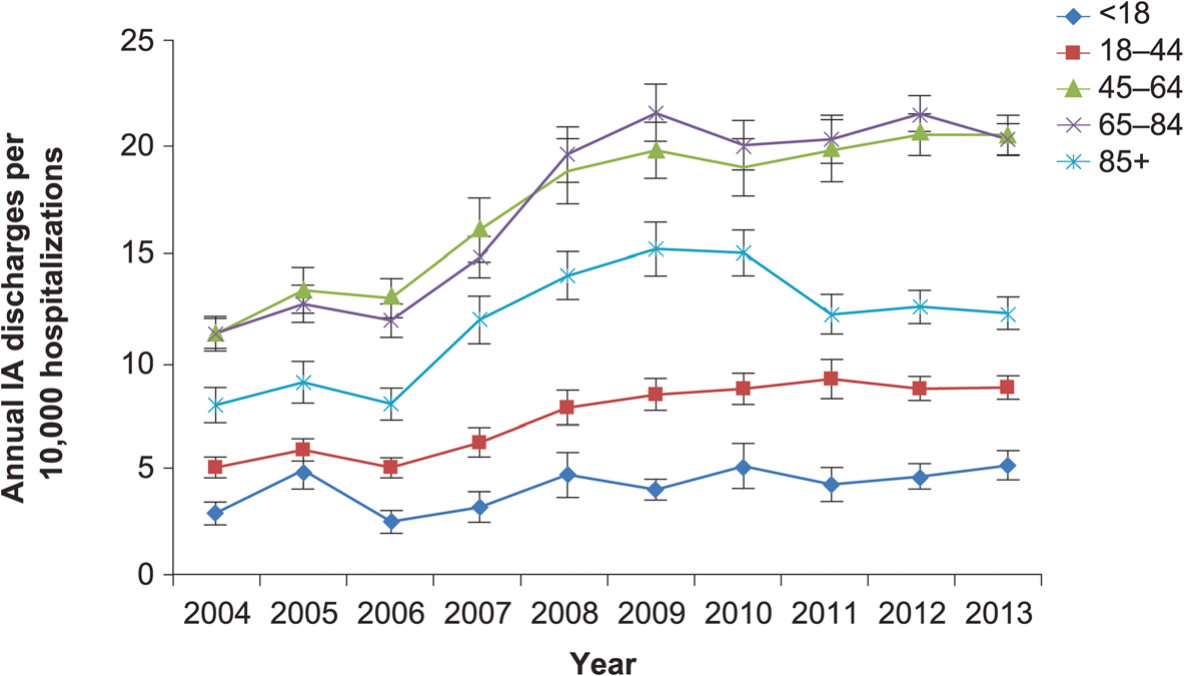
Annual incidence of hospitalizations (per 10,000 hospitalizations) with invasive aspergillosis, by age stratum. IA, invasive aspergillosis; vertical bars represent standard error

In contrast to population incidence, when case volume was standardized to the annual total hospitalizations in the US, the incidence of IA hospitalizations was highest (and similar to one another) in the 45–64 and 65–84 years of age groups, with the oldest age group occupying the middle of the incidence distribution in each year (Fig. 4). The steepest rise in this incidence occurred between 2006 and 2009 with subsequent stabilization (Fig. 4).

When examining the volume of IA cases by geographic area over the observation period, the South emerged as the region with the highest number of cases as well as the steepest rise in cases over time (Table 1). Despite this imbalance in absolute volume, the annual incidence in each of the regions was similar (Table 1). The incidence of IA standardized to total hospitalizations exhibited similar patterns (Table 1).

**Table 1:** Annual hospitalizations with a diagnosis of invasive aspergillosis, by region

	2004		2005		2006		2007		2008		2009		2010		2011		2012		2013	
	N	SE	N	SE	N	SE	N	SE	N	SE	N	SE	N	SE	N	SE	N	SE	N	SE
Volume																				
NE	5279	567	5777	653	5864	572	7899	1293	9343	1431	9082	1048	10,439	1512	10,354	1801	9560	701	9070	696
MW	7252	979	8241	1112	5974	709	7596	911	10,417	1355	12,024	1457	11,755	1539	11,061	1521	11,175	827	10,995	795
S	11,074	1055	11,562	1020	12,228	1238	14,785	1867	20,440	2551	18,647	1379	21,150	1740	20,912	1949	22,155	1338	20,580	1076
W	6169	621	10,053	1410	8296	1001	10,467	1218	12,092	1311	15,324	2202	9826	916	11,198	1116	11,125	727	11,235	729
Incidence per 100,000 population																				
NE	9.83	1.06	10.92	1.23	10.71	1.04	14.45	2.36	17.01	2.61	16.54	1.91	18.86	2.73	18.65	3.24	17.14	1.26	16.21	1.24
MW	11.19	1.51	12.85	1.73	9.02	1.07	11.44	1.37	15.65	2.04	18.06	2.19	17.55	2.3	16.47	2.26	16.6	1.23	16.28	1.18
S	10.69	1.02	11.06	0.98	11.21	1.13	13.39	1.69	18.3	2.28	16.69	1.23	18.41	1.51	18.02	1.68	18.89	1.14	17.38	0.91
W	9.31	0.94	15.06	2.11	11.96	1.44	14.93	1.74	17.07	1.85	21.63	3.11	13.62	1.27	15.37	1.53	15.12	0.99	15.13	0.98
Incidence per 10,000 all-cause hospitalizations																				
NE	7.07	0.76	7.88	0.89	7.94	0.77	10.75	1.76	12.68	1.94	12.42	1.43	14.5	2.1	14.54	2.53	13.69	1	13.48	1.03
MW	8.44	1.14	9.45	1.28	6.81	0.81	8.69	1.04	11.84	1.54	13.96	1.69	13.88	1.82	13.2	1.81	13.56	1	13.74	0.99
S	7.72	0.74	7.97	0.7	8.39	0.85	10.11	1.28	14.03	1.75	12.95	0.96	14.81	1.22	14.78	1.38	15.7	0.95	14.89	0.78
W	8.69	0.87	13.8	1.94	11.29	1.36	14.06	1.64	16.18	1.75	20.66	2.97	13.28	1.24	15.33	1.53	15.56	1.02	15.95	1.03

*N* number of annual hospitalizations, *SE* standard error, *NE* Northeast, *MW* Midwest, *S* South, *W* West

**Fig. 5:**
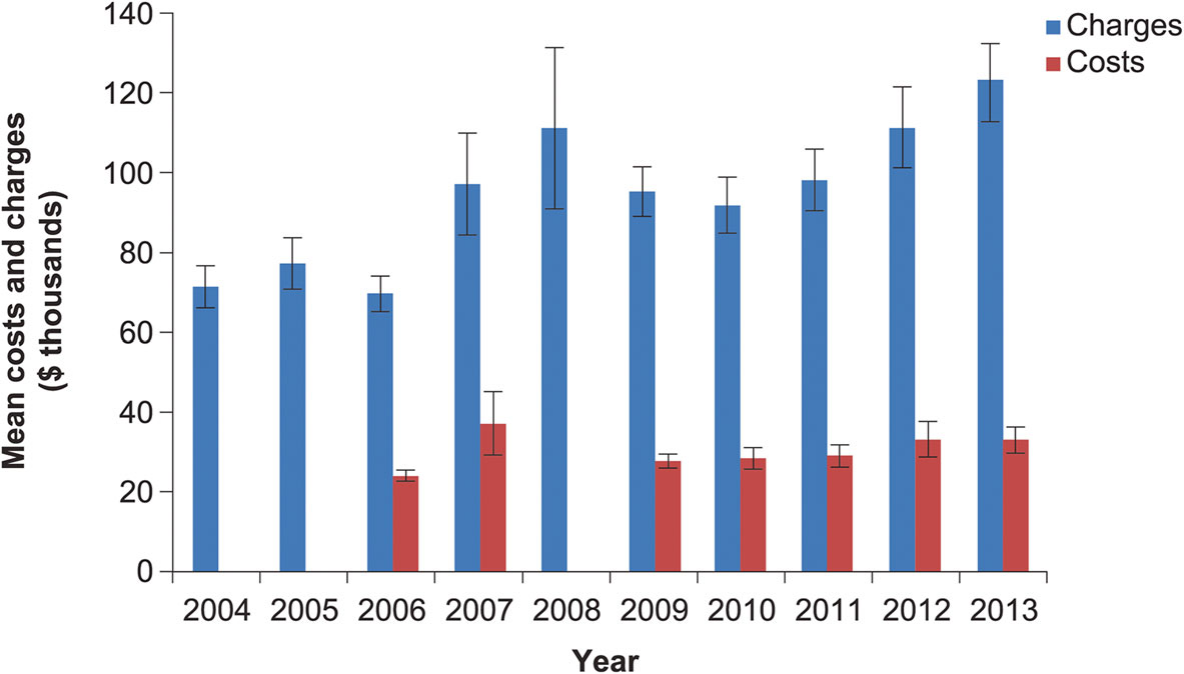
Hospital charges and costs for principal diagnosis of invasive aspergillosis*. Vertical bars represent standard error. *Costs unavailable for years 2004, 2005 and 2008. Dollar values approximate actual costs of services, as calculated through application of the Medicare cost-to-charge ratio

**Fig. 6:**
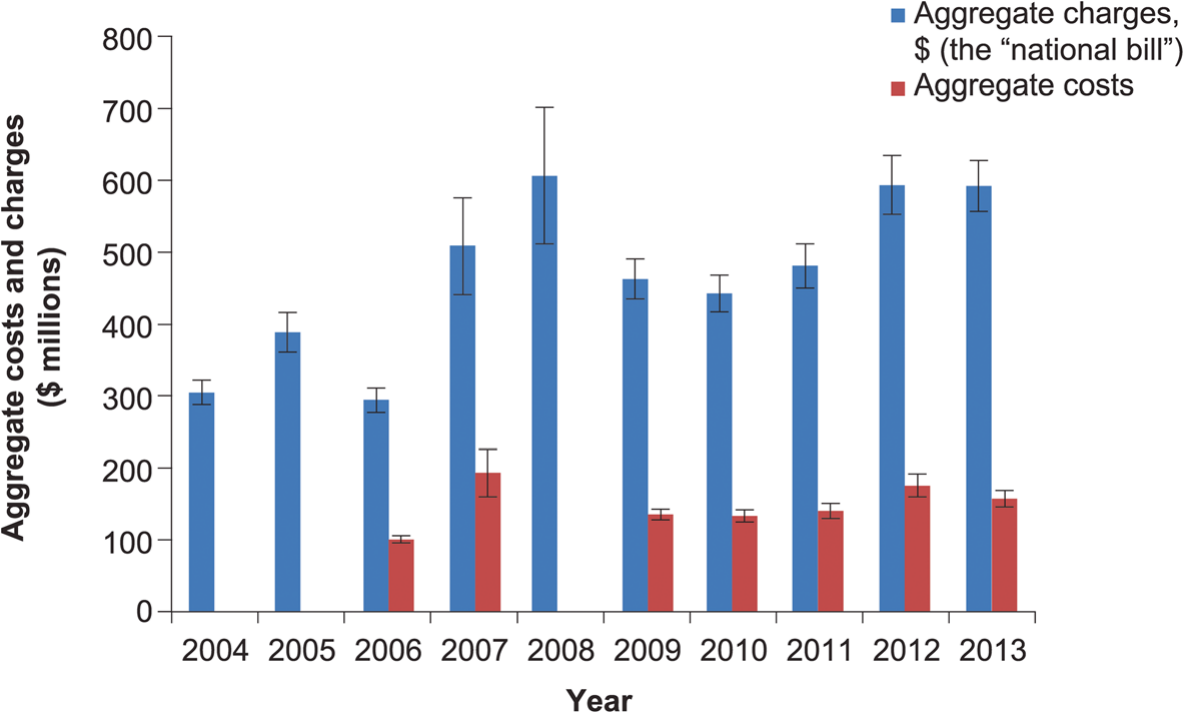
Aggregate hospital charges (“national bill”) and costs for principal diagnosis of invasive aspergillosis*. Vertical bars represent standard error. *Costs unavailable for years 2004, 2005 and 2008. Dollar values represent the hospital’s billed charges to payors

As a proportion of all IA hospitalizations, those where IA was the principal diagnosis dropped over the period of the study from 14.4% in 2004 to 9.3% in 2013. Hospital mortality rose from 10 to 12% over the timeframe of the study. While mean hospital LOS decreased from 13.3 (SE 0.07) days in 2004 to 11.5 (SE 0.6) days in 2013, the corresponding mean hospital charges rose from $71,164 (SE $5248) to $123,005 (SE $9738) (Fig. 5). After adjusting for inflation, this rise was more modest but still substantial, going from $101,748 (SE $7504) in 2004 to $123,005 (SE $9738) in 2013. Hospital costs showed greater stability over time than the corresponding charges, rising only modestly from $23,729 (SE $1346) in 2006 to $32,700 (SE $3378) in 2013 (Fig. 5). After adjusting for inflation, the costs essentially remained stable: $32,154 (SE $1824) in 2006 and $32,700 (SE $3378) in 2013. The case was similar for aggregated charges and aggregated costs (Fig. 6).

## Discussion

We confirm an increase in IA cases among hospitalized patients in the decade from 2004 to 2013. The magnitude of growth in the age-adjusted annual volume of IA hospitalizations in the current study (4.4%) echoes that noted within the National Inpatient Sample (NIS) by Vallabhaneni and co-authors (2.9%) [3]. To build on those authors’ work, we examined in detail the changes in IA hospitalizations over time. We stratified the data by such important factors as age and geography, and these stratifications clarify the nuances of the fluctuations in the IA incidence over the time frame of the study, which are smoothed out when looking at the overall population [3]. Despite these fluctuations, however, there was general growth in volume, which neared 50% over the decade examined. Normalizing the annual incidence to all US hospitalizations adds further granularity to understanding the impact of IA over time at the hospital level. Finally, our study adds to the understanding of the hospital resource utilization burden associated with IA.

We further observed that the proportion of IA hospitalizations with IA as the principal diagnosis dropped from 14 to 9% during the study period. Both of these estimates are far below a prior analysis of the NIS, a part of the HCUP, in 1996, in which over one quarter of all IA admissions carried it as the principal diagnosis [4]. Although it is reasonable to assume that most IA requires inpatient treatment, the precipitous and continuing drop in IA as the primary reason for hospitalization we note implies that other maladies are taking precedence over IA in necessitating admissions. In other words, IA may no longer represent the primary driver for hospitalization.

Our finding of a rise in crude mortality from 10 to 12% among patients with IA as the principal diagnosis, if not a cause for concern, at the very least requires further exploration. This is particularly necessary since analyses of the same source have consistently reported the death rate in this subgroup of 14 to 15% [4, 20]. It is likely that the explanation for the increase in mortality that we observed is due to the parallel increase in the severity of underlying illness among groups of patients whose diseases are now treated more aggressively and routinely, resulting in heightened susceptibility to IA. However, this requires confirmation.

We also found that while the IA-associated LOS decreased, the inflation-adjusted charges attributed to it rose by over $20/admission between 2004 and 2013. However, despite this increase in charges, the costs remained stable at approximately $32,000. This disparity between charges and costs may be indicative of a widening gap between expenditures and reimbursement incurred by hospitals over time. Of equal importance, the national bill for hospitalizations with IA as the principal diagnosis nearly doubled from a little over $300 million in 2004 to nearly $600 million in 2013. Not unexpectedly, these charges far exceed those noted in 1996 ($112.5 million) [4]. Notably, in Dasbach and colleagues’ study, patients with IA as the primary diagnosis (27% of all IA patients) contributed approximately 18% of the total aggregate US costs associated with all IA hospitalizations [4]. This implies that, given the 9% prevalence of IA as the principal diagnosis relative to all hospitalizations involving an IA code, and the total national bill for IA as principal diagnosis of approximately $600 million, the aggregate annual US charges for all IA-related hospitalizations in 2013 are likely exceeded $6 billion.

Our findings point to year-to-year and geographic variations in IA hospitalization incidence. Although our data do not allow for examinations of causality, these results point to potential directions for future research to shed light on whether there are modifiable risk factors and preventive strategies that may lessen the burden of IA hospitalizations.

Our study has a number of limitations. We may have overestimated the number of IA cases when all ICD-9-CM codes were added together, as any overlaps in these diagnoses within a single hospitalization cannot be identified in the HCUPnet. Though unavailable to us in the current analysis, in a separate study in this population, we reported the crude 30-day readmission rate to be 18% among survivors of a prior IA hospitalization [20]. Similarly, the annual incidence of IA may also be overestimated, as all cases were counted as first occurrences, when in fact a certain proportion of them may have represented readmissions. Additionally, the HCUPnet data lack the granularity necessary to examine important clinical, demographic and hospital factors that may explain some of our findings. The strength of the current study, however, lies in its vast numbers and broad generalizability to all US institutions.

## Conclusions

Invasive aspergillosis is a substantial problem in the US, with an age-adjusted annual growth rate of over 4% between 2004 and 2013. We observed a 2% rise over time in IA-associated mortality. However, in the current dataset we could not confirm whether this is indeed due to confounding by increased illness severity and/or treatment intensity. Therefore, this finding requires further scrutiny. Additionally, there is a widening gap between costs and reimbursements for IA hospitalizations, which may present an increasing strain on hospital resources. Given its high and growing morbidity, mortality and costs, intensive measures to prevent IA remain a necessity.

## Authors’ information

Rachel Harrington was affiliated to Astellas Pharma Global Development, Inc., Northbrook, IL, USA at the time of the study.

## Abbreviations

AHRQ: Agency for Healthcare Research and Quality
HCUP: Healthcare Cost and Utilization Project
HCUPnet: Healthcare Cost and Utilization Project Network
IA: Invasive aspergillosis
ICD: International Classification of Diseases
LOS: Length of stay
NIS: National Inpatient Sample
SE: Standard error
